# Adult-type anomalous origin of the left coronary artery from the pulmonary artery and right coronary-right atrial fistula: a case report

**DOI:** 10.1186/s12872-023-03686-x

**Published:** 2024-01-05

**Authors:** Hao Luo, Ofe Eugene Kwaku, Yinglong Lai, Rongchuan Yue

**Affiliations:** 1 Department of Cardiology, Affiliated Hospital of North Sichuan Medical College, Nanchong, China; 2 Department of Cardiology, People’s Hospital of Guang ’an District, Guang ’an, 638550 China; 3 Department of Cardiac and vascular surgery, Affiliated Hospital of North Sichuan Medical College, Nanchong, China

**Keywords:** ALCAPA, Congenital heart disease, Coronary artery fistula, Surgical intervention

## Abstract

**Background:**

Anomalous origin of the left coronary artery from the pulmonary artery (ALCAPA) is a rare congenital cardiac anomaly, mortality rates in infancy reach approximately 90%, with only a small number of patients surviving into adulthood, therefore, most of the literature reports mainly focus on infantile type.

**Case presentation:**

A 55-year-old female was admitted due to persistent repeated chest pain experienced and had worsened for unknown reasons. Color doppler echocardiography, coronary computed tomographic angiography, and coronary angiography confirmed the diagnosis of ALCAPA and concurrent right coronary artery-right atrial fistula. The symptoms of chest pain exhibited notable improvement subsequent to corrective surgery for the anomalous origin of the coronary artery.

**Conclusions:**

This report shows an unique case of ALCAPA in an adult patient, characterizing the condition’s combination with a right coronary-right atrial fistula, and it is prone to misdiagnosis and misdiagnosis. We aim to provide valuable insights for clinical diagnosis and treatment of ALCAPA.

## Background

Bland-White-Garland (BWG) syndrome, or anomalous origin of the left coronary artery from the pulmonary artery is a rare congenital cardiac anomaly with an incidence rate of approximately 1/300,000, accounting for 0.25–0.5% of congenital heart diseases [1]. The presence of this disease was first reported by Brooks in 1885, while its clinical manifestation was described by Bland, White and Garland in 1933. Most ALCAPA patients exhibit no typical clinical symptoms. In addition, coronary artery fistula is also a congenital cardiovascular malformation, accounting for 0.2–0.4% of congenital heart diseases, and it often originates from the right coronary artery or its branches [2]. The right coronary artery-right atrial fistula is one of them and is even rarer. It is extremely rare for a patient such as the one reported in this case to survive up to 55 years without surgical treatment.

## Case presentation

**Fig. 1:**
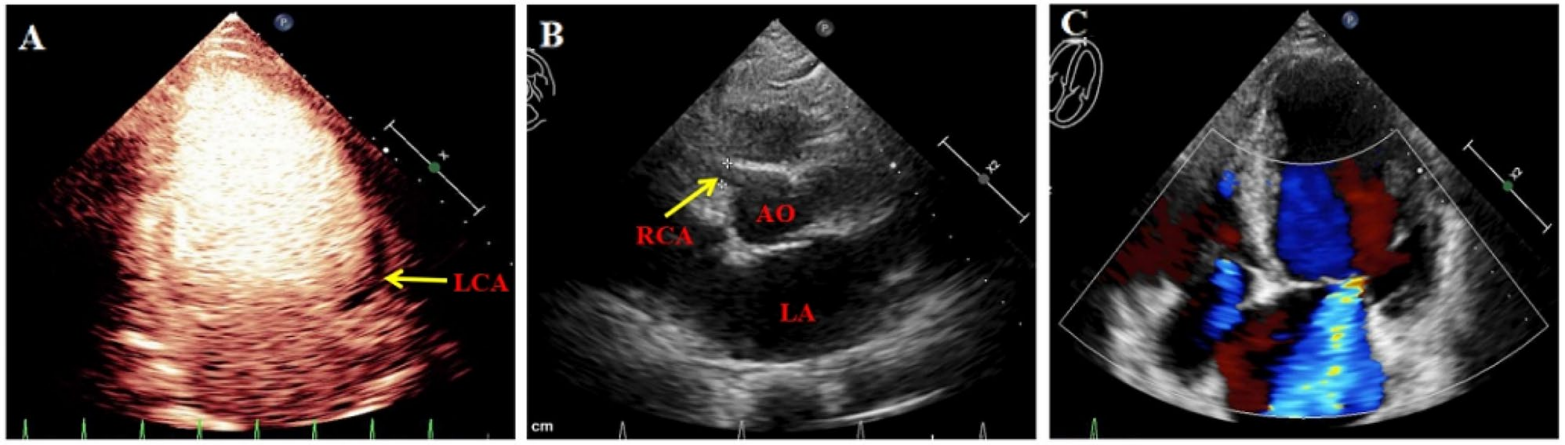
Preoperative color Doppler echocardiography. **A** The left coronary artery (yellow arrow) visible in the left posterior lateral wall of the middle part of the pulmonary artery. **B** Dilation of the right coronary artery (yellow arrow) originating from the right aortic sinus; **C**: Star point blood flow signal can be seen in the myocardium of ventricular septum and lateral wall of left ventricle, PW detection and diastolic phase are the main blood flow signal. AO: aorta, LA: left atrium, LCA: left coronary artery, RCA: right coronary artery

**Fig. 2:**
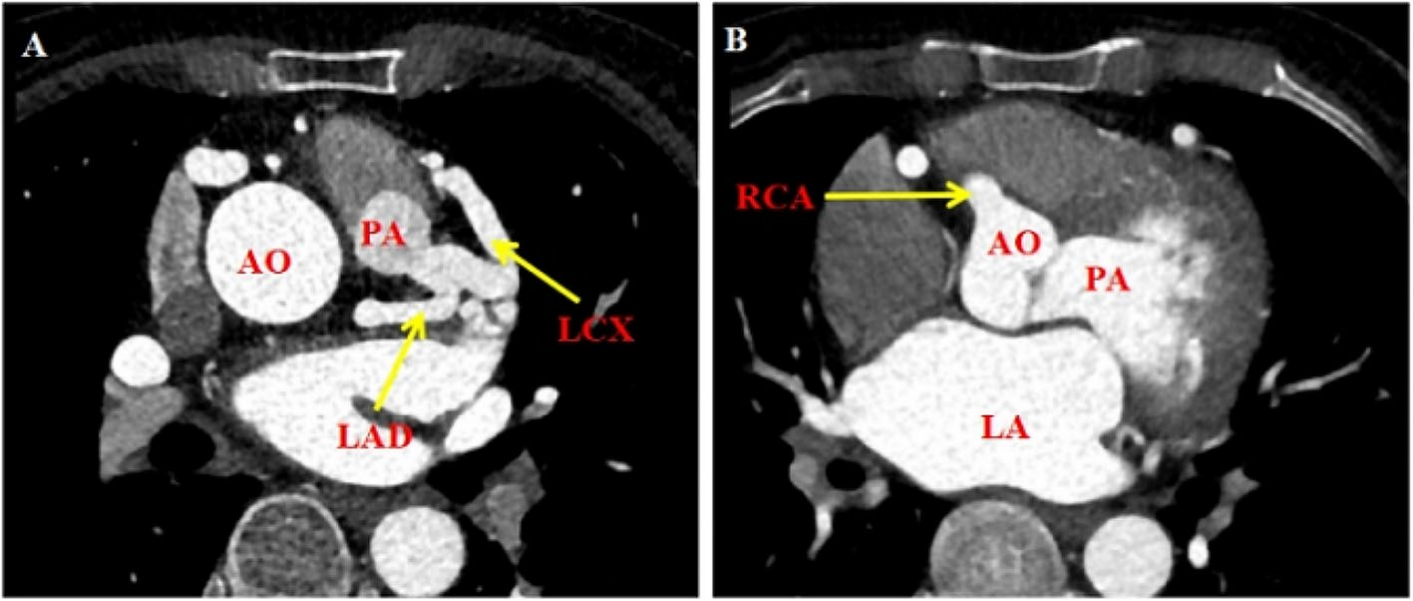
Preoperative coronary computed tomographic angiography. **A** The ectopic left coronary artery originated from the pulmonary artery. **B** The right coronary artery originates from the right aortic sinus. AO: aorta, PA: pulmonary artery, LA: left atrium, LAD: left anterior descending branch, LCX: left circumflex branch, RCA: right coronary artery

**Fig. 3:**
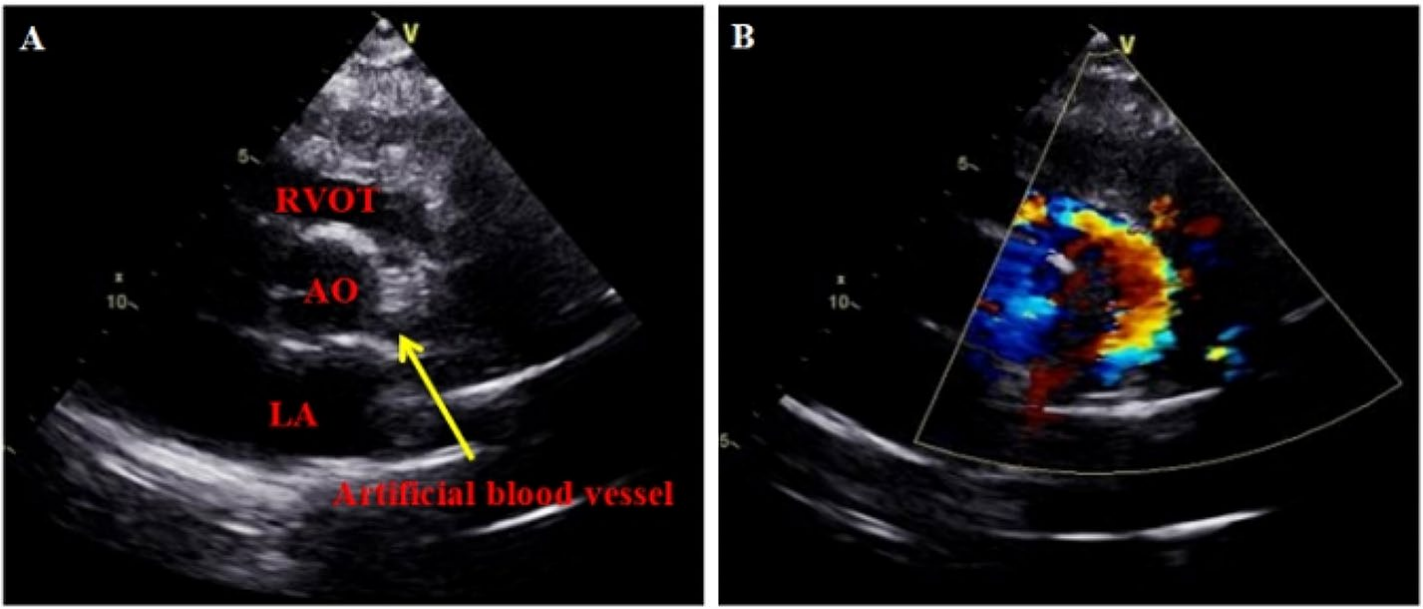
Postoperative color doppler echocardiography. **A** A 7 mm wide tubular echoless area - artificial blood vessel (yellow arrow) could be seen at about 4 o ‘clock on the short axis section of the great artery. **B** Color blood flow Doppler imaging indicated that star point blood flow signals could be seen in the myocardium of ventricular septum and left ventricular wall. AO: aorta, LA: left atrium, RVOT: right ventricular outflow tract, Artificial blood vessel: artificial blood vessel

The patient, a 55-year-old female, was admitted due to persistent chest pain experienced over a period of 2 years, and had worsened in the past 2 months. Both the patient and her family denied that she had a history of heart failure. Upon examination, the patient’s vital signs were stable, and no significant murmurs were detected in the heart. Electrocardiogram results showed the following: (1) sinus tachycardia; (2) suspected old myocardial infarction in the high lateral wall; and (3) T-wave changes. Color Doppler echocardiography findings (Fig. 1) showed enlargement of the left heart and mild enlargement of the right heart. Specifically, the measurements revealed a left ventricular end-diastolic diameter of 60 mm, a left atrial end-diastolic diameter of 47 mm, a right ventricular end-diastolic diameter of 23 mm, and a right atrial end-diastolic diameter of 40 mm. Other abnormalities observed during the echocardiography included dilation of the right coronary ostium, an abnormal side branch of the left posterior wall of pulmonary artery, apical thinning, the reduced motion of the left ventricle, and moderate to severe Carpentier-type I mitral valve regurgitation. The results of coronary angiography showed no visible left main stem, a diffuse large tumor-like dilation that spread throughout the periphery of the right coronary opening, and an evident lateral branch circulation in the right coronary opening resembling a normal tube diameter towards the left coronary. A large tumor-like expansion was observed in the branch circulation from the right coronary periphery towards the left coronary artery. An atrial fistula is present in the conical branch of the right coronary artery. No significant plaques or stenosis were found in either the left or right coronary arteries. Coronary computed tomographic angiography (CCTA, Fig. 2) demonstrates rightwards dominance, an anomalous origin of the left coronary artery from the pulmonary artery, and an origin of the right coronary artery from the right coronary sinus. Both the right and left coronary arteries, along with their branches, exhibit numerous twists and dilations. The diagnosis includes an anomalous origin of the left coronary artery from the pulmonary artery, a fistula between the right coronary artery and the right atrium, and a coronary artery aneurysm. After admission, the patient received treatments aimed at improving the overall functioning of the heart, such as diuretics, and circulatory enhancers. The patient underwent corrective surgery for the anomalous origin of the coronary artery and aortocoronary bypass under extracorporeal circulation. The procedure involved routine cannulation of the ascending aorta for blood supply, as well as cannulation of the superior and inferior vena cava for blood drainage, and insertion of the left heart drainage tube into the right upper pulmonary vein to establish extracorporeal circulation. Y-tubes were inserted into the aortic root and pulmonary artery root. The aortic root and bilateral pulmonary arteries were blocked, cardioplegia solution was perfused to the aortic root and the pulmonary artery root. Ice chips were placed on the surface of the heart surface to successfully arrest it. A T-cut was made in the pulmonary artery, releasing the left coronary artery. The left coronary artery was then clipped with button scissors, and a GORE 6 mm artificial blood vessel was anastomosed to the side of the left coronary with a 6 − 0 CV line. A bovine pericardial patch of appropriate size was successfully used to patch the pulmonary artery defect. The patch was secured with a continuous suture technique using 5 − 0 prolenesuture, effectively closing the pulmonary artery incision. Additionally, a hole was created at the aortic root, and the GORE 6 mm artificial blood vessel, was carefully positioned in front of the pulmonary artery. Anastomosis of the artificial blood vessel to the aortic root was performed using a 6 − 0 CV suture, to ensure thorough hemostasis. During the surgical procedure, the patient experienced an episode of cardiac fibrillation. Prompt intervention in the form of cardiac massage and defibrillation successfully restored the patient’s heart rhythm to sinus rhythm. After achieving stable circulation, the machine was turned off, and the tube was removed. Following the surgical intervention, the patient showed a significant improvement in anterior chest pain compared to the pre-operative state. A subsequent color Doppler echocardiogram revealed a successful connection between the left coronary artery and the aortic root via the artificial blood vessel (Fig. 3).

## Discussion and conclusion

Depending on the degree of collateral circulation between the coronary arteries, it can usually be divided into infant and adult types. In infants, the left and right coronary arteries do not form enough collateral circulation, and the early decrease in pulmonary vascular pressure leads to insufficient blood supply to the left coronary artery, easily causing myocardial ischemia and significant hypoxia symptoms, often accompanied by reduced left ventricular function. Patients often die from myocardial infarction and heart failure [3]. The adult type accounts for about 10%. The left and right coronary arteries can compensate by thickening and establishing a rich collateral circulation, increasing blood flow and reducing the degree of myocardial ischemia. However, due to the gradually decreasing resistance of the pulmonary circulation and pressure in the pulmonary artery, blood flow in the right coronary artery easily flows from the left coronary artery into the pulmonary artery through the rich collateral circulation, creating a “coronary artery to pulmonary artery steal phenomenon”, which is often associated with myocardial damage and easily causes angina pectoris, arrhythmias, and sudden cardiac death [4]. For the adult type ALCAPA, the collateral circulation formed between the two coronary arteries largely determines the prognosis and long-term survival of the patient. Even without any symptoms, sudden death can occur, and about 90% of patients suddenly die at an average age of 35 years [5]. Adult ALCAPA symptom often atypical and is often an incidental finding, Esfehani reported a patient who was admitted for atypical chest pain and underwent surgical treatment in 2017, and postoperative relief of chest pain was obvious [6], and Wang et al. report an adult case with asymptomatic ALCAPA and anterior mitral leaflet prolapse, then underwent successful reimplantation of the left coronary artery into the aorta [7].

**Fig. 4:**
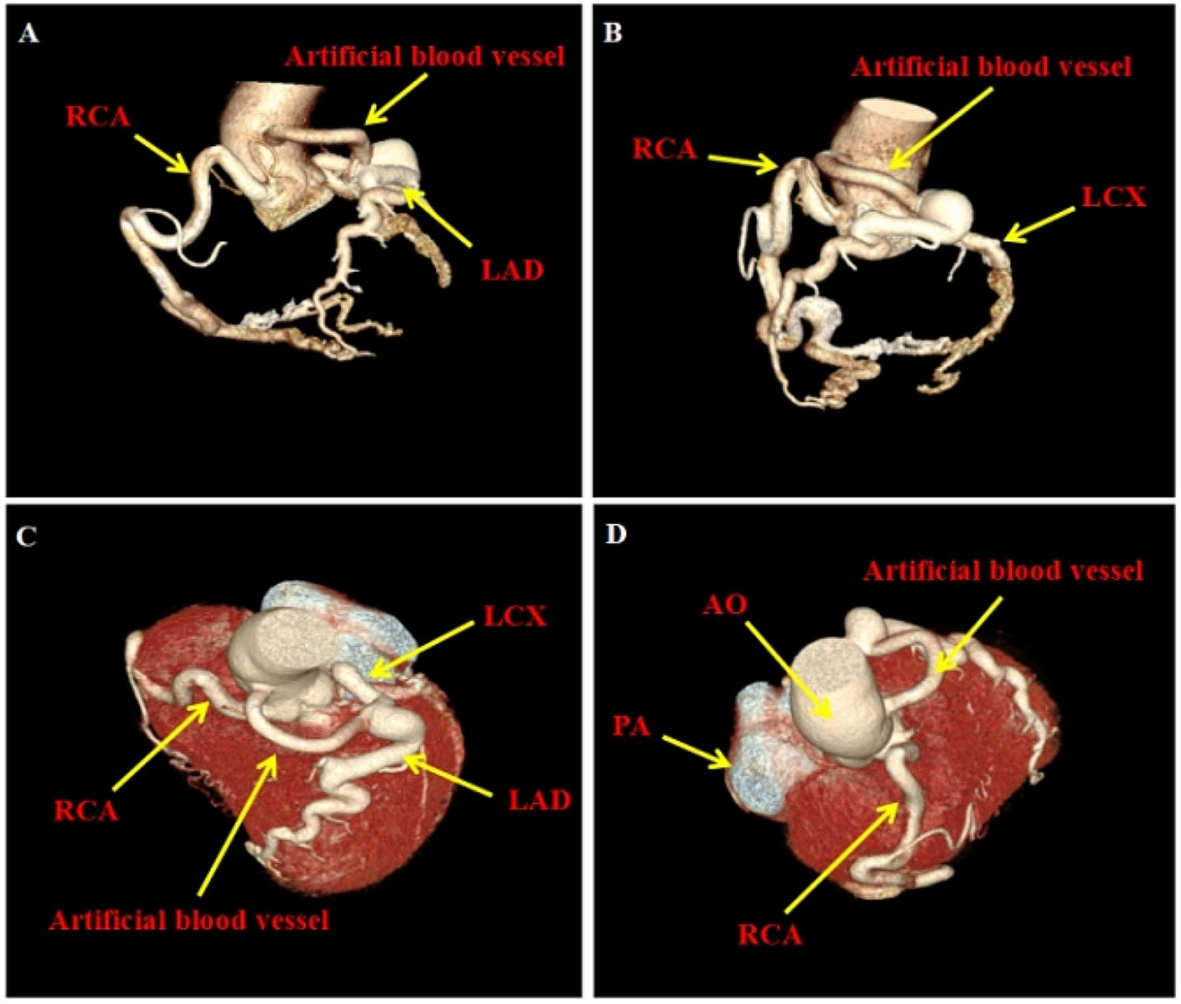
Postoperative coronary computed tomographic angiography. **A-D** The 6 mm GORE-TEX artificial vessel was connected to the left coronary artery and the aortic root for coronary branch dilation, and the coronary artery presented a right dominant type. AO: aorta, PA: pulmonary artery, LAD: left anterior descending branch, LCX: left circumflex branch, RCA: right coronary artery, Artificial blood vessel: artificial blood vessel

In order to prevent the development of cardiac insufficiency, and reduce the risk of sudden cardiac death, corrective surgery has become an effective treatment for ALCAPA syndrome [8]. Therefore, adult patients with ALCAPA, regardless of clinical manifestations and whether there are complications, should undergo timely surgery intervention. The patient was admitted to the hospital due to recurrent chest pain, with no prior history of cardiovascular disease. The high sensitive cardiac troponin level was not high (0.011 ng/mL). Echocardiography revealed dilation of the proximal right coronary artery with an inner diameter of approximately 9 mm, and a duct was noted branching from the lateral wall of the middle segment of the pulmonary trunk. Coronary angiography showed a markedly tortuous and dilated right coronary artery with huge tumor-like dilatation, rich collateral circulation, and a right coronary-conical branch atrial fistula, but the opening position of the left coronary artery was not visualized. CCTA, as a non-invasive examination method, can clearly display the collateral circulation between the left and right coronary arteries. Compared to coronary angiography, it can also accurately and intuitively visualize the origin and course of coronary arteries. In addition, it can depict the anatomical relationships of the great vessels and provide high-resolution three-dimensional reconstruction images. The European Society of Cardiology has recommended CCTA as the preferred diagnostic method for coronary artery origin abnormalities [9, 10]. The patient underwent CCTA, which confirmed multiple twists and dilations of the right coronary artery, the left coronary artery and its branches, and the anomalous origin of the left coronary artery from the pulmonary artery. According to the patient’s medical history, cardiac symptoms, and comprehensive imaging findings, the patient was diagnosed with ALCAPA and a right coronary artery-right atrial fistula. CCTA has shown its superiority in diagnosing these rare cases. Physicians should have a high index of suspicion for such rare coronary abnormalities, especially when patients present with recurrent chest pain without any obvious causes. Early detection and surgical intervention can significantly improve the prognosis and quality of life of these patients. In recent years, coronary artery transplant surgery has gradually become the primary surgical method for treating anomalous origins of the left coronary artery from the pulmonary artery due to its minimal operative complications, physiological anatomical compliance, superior post-operative results, low rate of complications, and high long-term patency rate of the coronary artery [11]. Consequently, on exclusion of surgical contraindications, we performed surgery to correct the anomalous origin of the coronary artery and performed coronary artery bypass graft surgery. The patient showed significant improvement in anterior chest pain post-operation. A follow-up CCTA after one month revealed (Fig. 4): The coronary artery was right dominant, the left coronary artery showed postoperative changes, the artificial blood vessel originated from the aortic root and was connected to the left coronary artery trunk, with the right coronary artery originating from the right coronary sinus. Although this procedure addresses the difficult re-implantation of the left coronary artery on the basis of achieving double coronary artery anatomy and physiology, the presence of an artificial vessel may lead to pulmonary artery stenosis and thrombosis in the artificial vessel, and the long-term prognosis of this surgical modality is unknown [12].

The clinical manifestations of ALCAPA lack specificity, making misdiagnosis and missed diagnosis quite likely. The middle-aged and elderly patient reported in this article was confirmed to have an anomalous origin of the left coronary artery from the pulmonary artery and a concurrent right coronary artery-right atrial fistula through cardiac Doppler echocardiography, coronary artery CT angiography, and coronary angiography. This is a rare occurrence worldwide. Surgical intervention is the most effective treatment for Bland-White-Garland syndrome combined with a right coronary artery-right atrial fistula. The patient in this case declined fistula repair surgery, so we temporarily performed surgery to correct the anomalous origin of the coronary artery and coronary artery bypass graft surgery. The patient’s anterior chest pain symptoms disappeared shortly after surgery, and cardiac function improved. However, the long-term effect awaits further follow-up.

## Data Availability

All relevant data supporting the conclusions of this article are included within the article.
